# A Cross-Sectional Study of Avian Influenza in One District of Guangzhou, 2013

**DOI:** 10.1371/journal.pone.0111218

**Published:** 2014-10-30

**Authors:** Haiming Zhang, Cong Peng, Xiaodong Duan, Dan Shen, Guanghua Lan, Wutao Xiao, Hai Tan, Ling Wang, Jialei Hou, Jiancui Zhu, Riwen He, Haibing Zhang, Lilan Zheng, Jianyu Yang, Zhen Zhang, Zhiwei Zhou, Wenhua Li, Mailing Hu, Jinhui Zhong, Yuhua Chen

**Affiliations:** 1 Guangzhou Animal Disease Prevention and Control Center, Guangzhou, China; 2 Huadu Animal Health Inspection Institute, Huadu, China; National Institute for Viral Disease Control and Prevention, CDC, China, China

## Abstract

Since Feb, 2013, more than 100 human beings had been infected with novel H7N9 avian influenza virus. As of May 2013, several H7N9 viruses had been found in retail live bird markets (LBMs) in Guangdong province of southern China where several human cases were confirmed later. However, the real avian influenza virus infection status especially H7N9 in Guangzhou remains unclear. Therefore, a cross-sectional study of avian influenza in commercial poultry farms, the wholesale LBM and retail LBMs in one district of Guangzhou was conducted from October to November, 2013. A total of 1505 cloacal and environmental samples from 52 commercial poultry farms, 1 wholesale LBM and 18 retail LBMs were collected and detected using real-time RT-PCR for type A, H7, H7N9 and H9 subtype avian influenza virus, respectively. Of all the flocks randomly sampled, 6 farms, 12 vendors of the wholesale LBM and 18 retail LBMs were type A avian influenza virus positive with 0, 3 and 11 positive for H9, respectively. The pooled prevalence and individual prevalence of type A avian influenza virus were 33.9% and 7.9% which for H9 subtype was 7.6% and 1.6%, respectively. None was H7 and H7N9 subtype virus positive. Different prevalence and prevalence ratio were found in different poultry species with partridges having the highest prevalence for both type A and H9 subtype avian influenza virus. Our results suggest that LBM may have a higher risk for sustaining and transmission of avian influenza virus than commercial poultry farms. The present study also indicates that different species may play different roles in the evolution and transmission of avian influenza virus. Therefore, risk-based surveillance and management measures should be conducted in future in this area.

## Introduction

Human infection with H7N9 avian influenza virus was first reported in March in Eastern China [1]. As of 25 October 2013, 137 human cases were reported in 13 provinces with 45 deaths (http://www.who.int/influenza/human_animal_interface/influenza_h7n9/Data_Reports/en/index.html). The infections caused global concerns about the potential of the virus to start an influenza pandemic [2], [3] and have already caused huge economic losses for the poultry industry including trade in live poultry in China. However, while the highly pathogenic avian influenza A viruses (HPAIV), such as H5N1 and H7N7, often causes severe disease in poultry preceding human infections [4], [5], the novel H7N9 virus causes no or only mild clinical signs in poultry [6]. This means that the H7N9 virus is likely to spread silently in poultry. Although H7N9 viruses had been isolated from poultry and environments at the LBM [7], [8], the source of the infections remain unclear [9].

In addition, although H9N2 avian influenza virus is low pathogenic avian influenza virus (LPAIV) and usually causes mild disease or asymptomatic infection in poultry, the virus was considered having donated the internal genes to H5N1 in Hong Kong and H7N9 avian influenza virus [1], [10]. Since 1998, several human cases infected with H9N2 avian influenza virus have been reported [11], [12]. Thus, the epidemiological investigation of H9 subtype virus is crucial and necessary in southern China.

Southern China is considered as one of “influenza epicenters” worldwide [13], not only due to the amount of influenza outbreaks in both poultry and human, but also the fact that almost all the subtypes of avian influenza virus were isolated from this region. Contact between different species of poultry, other animals and humans facilitate the transmission and evolution of influenza viruses [14]. Furthermore, the habit of purchasing live poultry in this area increases the risk of human infection with avian influenza virus. Therefore, the epidemiological study of avian influenza is critically important in southern China. In this study, a cross-sectional study of type A, H7, H7N9 and H9 subtype AIV in poultry commercial farms, wholesale LBM and retail LBMs was conducted to figure out the infection status in Guangzhou, China. The results could be useful for government to plan and adjust the prevention and control strategies of avian influenza.

## Materials and Methods

All field studies were carried out in Huadu District of Guangzhou city, Guangdong province, China. No specific permission was required to test the farms and no endangered or protected species were involved. This study was conducted according to the animal welfare guidelines of the World Organization for Animal Health [15] and approved by Guangzhou Bureau of Agriculture. The activities of sample collection were permitted by the farmers involved.

A cross-sectional study was carried out in 52 poultry commercial farms, 1 wholesale LBM and 18 retail LBMs in Huadu district, Guangzhou of Southern China from October to November, 2013.

### Target population and sampling strategy

Huadu district is one of 12 districts of Guangzhou city. There are 238 commercial chicken farms, 663 commercial waterfowl farms, 30 commercial pigeon farms, 22 retail LBMs and one wholesale LBM with 15 vendors distributed in 13 towns of Huadu district. The farms with inventory of more than 1000 were regarded as commercial farms in the study. Farms, retail LBMs and vendors were considered as population or flock. The poultry such as chicken, waterfowl, pigeon in farm, retail LBMs and/or vendors was individual.

A two-stage plus stratified random sampling strategy was used for sampling. For commercial farms and the wholesale LBM, the farms or vendors within wholesale LBM were divided into three stratums according to three poultry species as chicken, waterfowl and pigeon. For retail LBMs, the stratums were named according to the species sold the retail LBMs at the time of sampling. For commercial farms or the wholesale LBM, the first level sampling units were farms or vendors within the LBM, the second level sampling units were the individual poultry. For retail LBMs, the first and second level sampling units were LBMs and the individual poultry, respectively.

### Sample size and sample collection

For the first and second stage, the model of estimate prevalence and detecting disease were used, respectively. The number of commercial farms to be selected were calculated with 30% expected prevalence, 20% absolute error and 95% confidence; the number of vendors within the wholesale LBM were calculated with 40% expected prevalence, 10% absolute error and 95% confidence; the number of retail LBMs were calculated with 50% expected prevalence, 10% absolute error and 95% confidence. For the second stage, the number of individual to be sampled was calculated with 20%, 30% and 30% expected prevalence, respectively. The test sensitivity and confidence were the same as 90% and 95%. The expected prevalence of farms, vendors and retail LBMs used were all according to our historical surveillance results and experts’ opinions.

In each selected farm and vendor, 15 and 10 cloacal swabs were collected respectively. In general, 10 cloacal swabs were sampled for each species per retail LBM. In addition, the environmental samples were preferentially selected from feces, wet and dirty areas such as water troughs, drains and bird slaughter areas in LBMs according to previous studies [16], [17].

Sterile cotton-tipped swabs were used to collect samples. Swabs were pooled by species of poultry and sample type (cloacal or environmental sample), up to five swabs per tube with approximately 2 ml aliquots of phosphate buffered saline (PBS). All samples were preserved in laboratory at −80°C until tested.

### Laboratory methods

RNA were extracted using 5×MagMAX 96 viral Isolation Kit (Ambion, TX, USA) and amplified with TaqMan AIV-M Reagents Kit (Ambion, TX, USA) for type A AIV firstly. The positive pools were then tested using real-time RT-PCR of H7, H7N9 and H9 subtype, respectively. The H7N9 virus was detected using real-time RT-PCR with the primers recommended by World Health Organization (WHO) (http://www.who.int/influenza/gisrs_laboratory/cnic_realtime_rt_pcr_protocol_a_h7n9.pdf?ua=1) and Super Script III Platinum one-step qRT-PCR (Invitrogen, CA, USA). The H7 and H9 subtype was detected using H7 and H9 subtype AIV real-time RT-PCR Kit (TaiTai, Shenzhen, China), respectively. A positive farm, vendor or retail LBM was defined as a flock that at least one pool was detected with type A AIV, H7, H7N9 or H9 subtype positive.

### Statistical analysis

Microsoft Excel (Microsoft, Redmond, WA, USA), Epi Info 6.0 software (Centers for Disease Control and Prevention, Atlanta, GA, USA) and the Ausvet pooled prevalence calculator (Sergeant, ESG, Epitools epidemiological calculators. AusVet Animal Health Services and Australian Biosecurity Cooperative Research Centre for Emerging Infectious Disease) were used for the descriptive and statistical analysis. The individual prevalence of AIV was estimated by the Ausvet pooled prevalence calculator and compared using chi-square test.

## Results

Fifty-two commercial poultry farms (including 19 chicken farms, 20 waterfowl farms and 13 pigeon farms), 13 vendors within wholesale LBM and 18 retail LBMs were chosen in the present study. All selected farms locate on all seven towns in Huadu district, Guangzhou, other than one town without farms. All 19 LBMs were distributed on every town in Huadu district, Guangzhou.

### Population distribution

The distribution of positive population for type A AIV and H9 virus is shown in Table 1. A total of 6 commercial farms, 12 vendors of wholesale LBM and all 18 retail LBMs had at least one sample pool that yielded AIV. For H9 subtype virus, 0 commercial farms, 3 vendors and 11 retail LBMs were positive. No farms, vendors and retail LBMs were positive for H7 and H7N9 subtype virus.

**Table 1:** Population prevalence of type A and H9 subtype AIV.

Type of population	No. of population	Type A AIV		H9 virus	
		Positive number	Prevalence (%) (95%CI)*	Positive number	Prevalence (%) (95%CI)*
Commercial farm	52	6	11.5(4.4–23.4)	0	0.0(0.0–6.9)
Vendor	13	12	92.3(64.0–99.8)	3	23.1(5.0–53.8)
Retail LBM	18	18	100.0(81.5–100.0)	11	61.1(35.8–82.7)
In total	83	36	–	14	–

AIV, avian influenza virus; LBM, live bird market.

*Prevalence at flock level.

### Individual distribution

The pooled and individual prevalence of type A AIV and H9 virus in different flocks is shown in table 2. The individual prevalence of type A AIV in commercial farms, vendors and retail LBMs were 1.2%, 18.2% and 18.7%, respectively. The individual prevalence of H9 subtype virus in commercial farms, vendors and retail LBMs were 0.0%, 2.6% and 3.7%, respectively. In addition, no pool with H7 or H7N9 virus positive was found. We investigated the sources of the poultry in the LBMs and found that most AIV and H9 virus positive poultry were from other wholesale LBMs in Guangzhou and other poultry farms of other areas with only several avian influenza virus positive poultry were traced back to local farms.

**Table 2:** Pooled and individual prevalence of type A and H9 subtype AIV.

Sample source	Type A AIV				H9 virus			
	No. of pools	No. of positive pools	Pooled prevalence (95%CI) (%)	Individual prevalence (95%CI) (%)^a^	No. of pools	No. of positive pools	Pooled prevalence (95%CI) (%)	Individual prevalence (95%CI) (%)^a^
Commercial farm	156	9	5.8(2.7–10.7)	1.2(0.5–2.2)	156	0	0.0(0.0–2.3)	–
Vendor	41	26	63.4(46.9–77.9)	18.2(11.9–26.0)	41	5	12.2(4.1–26.2)	2.6(0.8–5.9)
Retail LBM	104	67	64.4(54.4–73.6)	18.7(14.4–23.4)	104	18	17.3(10.6–26.0)	3.7(2.2–5.8)
In total	301	102	33.9(28.6–39.5)	7.9(6.5–9.5)	301	23	7.6(4.9–11.3)	1.6(1.0–2.3)

AIV, avian influenza virus; LBM, live bird market.

a prevalence estimated by Ausvet pooled prevalence calculator.

### Species distribution and effects

The pooled and individual prevalence of type A AIV and H9 virus in different species are shown in Table 3. The individual prevalence of type A AIV was highest in partridges as all of the three pools were positive, followed by environmental (16.0%), chicken (10.4%), waterfowl (6.1%) and pigeon (2.9%). The statistical analysis showed that there were significant associations between poultry species and the virus detection for both type A AIV and H9 subtype virus (p<0.01).

**Table 3:** Pooled and individual prevalence and prevalence ratio of type A AIV for different poultry species.

Sample source	No. of pools	Type A AIV				
		No. of Positive pools	Pooled prevalence (95%CI) (%)	Individual prevalence (95%CI) (%)^a^^,^ b	Prevalence ratio (95%CI) (%)^c^	p-value
Chicken	109	46	42.2(32.8–52.0)	10.4(7.6–13.7)	1	–
Waterfowl	100	27	27.0(18.6–36.8)	6.1(4.0–8.8)	0.58(0.43–0.79)	<0.01
Pigeon	58	8	13.8(6.2–25.4)	2.9(1.2–5.7)	0.28(0.19–0.40)	<0.01
Partridge	3	3	100.0(29.2–100.0)	*	–	<0.01
Environmental	31	18	58.1(39.1–75.5)	16.0(9.4–24.5)	1.54(1.22–1.93)	<0.01
In total	301	102	33.9(28.6–39.5)	7.9(6.5–9.5)	–	–

AIV, avian influenza virus;

*Individual Prevalence of partridge can’t be calculated by the Ausvet pooled prevalence calculator.

a prevalence was estimated by Ausvet pooled prevalence calculator.

b *p*<0.01.

c PR was calculated based on individual prevalence.

Our results showed that partridges had several times increased risk of prevalence of type A AIV compared with chicken, whereas waterfowl and pigeon had reduced risk (p<0.01). Besides, partridges had elevated risk of H9 subtype virus compared with chicken, whereas waterfowl and pigeon had reduced risk (p<0.01). The detailed information is shown in Table 3 and Table 4.

**Table 4:** Pooled and individual prevalence and prevalence ratio of H9 subtype virus for different poultry species.

Sample source	No. of pools	H9 virus				
		No. of positive pools	Pooled prevalence (95%CI) (%)	Individual prevalence (95%CI) (%)^a^^,^ b	Prevalence ratio (95%CI)^c^	p-value
Chicken	109	16	14.7(8.6–22.7)	3.1(1.8–5.0)	1	–
Waterfowl	100	3	3.0(0.6–8.5)	0.6(0.1–1.8)	0.19(0.09–0.42)	<0.01
Pigeon	58	1	1.7(0.04–9.2)	0.3(0.0–1.9)	0.10(0.04–0.25)	<0.04
Partridge	3	2	66.7(9.4–99.2)	19.7(2.0–61.6)	6.35(4.66–8.67)	<0.01
Environmental	31	1	3.2(0.08–16.7)	0.7(0.0–3.5)	0.23(0.11–0.47)	<0.01
In total	301	23	7.6(4.9–11.3)	1.6(1.0–2.4)	–	–

a Prevalence was estimated by Ausvet pooled prevalence calculator.

b *p*<0.01.

c PR was calculated based on individual prevalence.

## Discussion

Guangzhou, the capital of Guangdong province, is located in southern China which is regarded as one of the epicenters of influenza [13]. The high-density of pigs, human and poultry with different species such as chicken, duck, goose, and pigeon was regarded as having facilitated the evolution of avian influenza virus [18]. In May 2013, one H7N9 avian influenza virus was detected from chicken sample in a retail LBMs in Guangzhou. Subsequently, several human H7N9 cases were confirmed in other cities of southern China. Therefore, the real infection status of avian influenza virus especially the H7N9 in this area needs to be investigated. To our knowledge, this is the first study carried out to detect the presence of avian influenza virus with random sampling in poultry industry including commercial poultry farms, wholesale LBM and retail LBMs in southern China.

This study identified the distinct infection variation between commercial farms and LBMs with respect to prevalence of different avian influenza subtypes. The low prevalence of type A AIV and no positive poultry sample of H9 virus from commercial farms, combined with consistent historically surveillance testing results, may indicate that type A AIV and H9 virus are not persisting in a majority of farms with more than 1000 inventory per farm in this region. In addition, several H9 viruses were found in LBMs including the wholesale LBM and retail LBMs, which was differed from commercial farms. We hypothesized that the poultry with H9 viruses were not from the local farms but from other districts, other cities or other provinces through live poultry trade. As the H9N2 subtype virus likely made the previous and ongoing contribution to the evolution of H7N9 virus [19], the probability of LBMs playing an important role in the evolution of H7N9 virus could not be excluded. Results of this study also posed a significant risk to the commercial poultry and even human beings as LBMs playing a crucial role in maintenance, amplification, and dissemination of avian influenza viruses as well [20]. The rest day and biosecurity measures have not eliminated the viruses but that it is worth further studies to assess the extent to which the rest day reduces the levels of viral contamination. In addition, no positive results of H7N9 subtype virus for all swabs might imply the low risk of human infection with H7N9 virus. However, the possible introduction of H7N9 virus through the live poultry trade as reported in other places [21] should not be neglected because the first human case of H7N9 in Guangzhou was reported in January 2014 and two positive retail markets were found in 2014 in Huadu district of Guangzhou [22].

Data in this study showed that type A and H9 subtype AIV were mostly frequently isolated from chickens, partridges and environmental samples. Differences in prevalence between different poultry species may be due to poultry source, representativeness of samples and replication pattern of different lineages of avian influenza virus [23]. And as H9N2 avian influenza virus had been reported to infect human beings and donated the internal genes to the novel H7N9 avian influenza virus, the high prevalence of H9 subtype in the present study may indicate the high risk of human infection with H7N9. For waterfowls including ducks and geese we studied, although the prevalence of H9 virus was low, the role of domestic ducks in the influenza virus ecosystem should not be neglected because the samples were all collected from apparently healthy domestic waterfowls and the prevalence of AIV was relatively high. Additionally, it was interesting to note that the prevalence of AIV and H9 virus for partridges were higher than other species and previous research [24]. Minor poultry were regarded to play an important role in the epidemiology of avian influenza viruses partly because of slower turnover rate than chickens and ducks which means they would have time to complete at least one full virus replication cycle of AIV. Moreover, as H9N2 isolate found in another minor poultry (quail) was identified the donor of the internal genes of H5N1 avian influenza virus that caused human disease in Hong Kong in 1997 [25], the role of partridge in evolution and ecology of the highly pathogenic avian influenza virus was not clear yet and needed further research.

In addition to cloacal samples, environmental samples were also studied. Previously, most studies focused on testing live birds rather than environmental samples in LBMs [26]–[28] with a few research focused on environmental samples [17], [29]. Interestingly, some environmental samples from some retail LBMs or vendors of wholesale LBM instead of the cloacal swabs were found to be negative indicating that the virus may not be presented in the market at the time samples were collected. In addition, the relatively high prevalence of type A AIV in environmental samples from LBMs meant that the avian influenza viruses could persist longer to infect the new susceptible poultry and transmit to other LBMs even farms through live poultry, trucks or/and other tools. Thus, the stricter biosecurity measures along with reasonable surveillance should be imposed immediately.

In conclusion, we reported that the LBMs including wholesale LBM and retail LBMs were the high-risk places for persisting and transmission of avian influenza virus. Different poultry species had different prevalence for type A AIV and H9 subtype virus with different PR shown above. Therefore, more appropriate and effective management measures are needed to reduce their potential threats to animal and human health. The high prevalence of type A AIV and relatively low prevalence of H9 subtype virus with no positive for H7 and H7N9 subtype virus warrant further investigation such as characterizing other subtypes of avian influenza virus which is crucial for developing a full understanding of AIV in southern China. The main focus of this study was viruses of the H7 and H9 subtypes and therefore further characterization of other influenza A viruses was not undertaken. Our study also highlights the need for coordinated actions between public health and veterinary services. Interventions that could effectively reduce the prevalence of avian influenza virus would benefit not just for poultry keepers but also the public in Guangzhou.

### Limitations

Our target population was confined to one district of Guangzhou mainly because husbandry practices and infection status of avian influenza virus are similar with other places in Guangzhou even Guangdong province. Additionally, backyard poultry farms were not included because the sampling frame was not clear. Nevertheless the absence of detection of H7N9 viruses in any samples from markets suggests that they may not have been infected at this time. Some selection bias may have occurred in retail markets because the composition of the species in the markets at the time of collection was based on the species present at the time and reflected stock remaining for sale at that time. Serological tests were not performed in this study as the main focus was on active infection in farms and markets. Subsequent tests performed by Provincial and National veterinary authorities and reported in the Ministry of Agriculture Official Veterinary Bulletin demonstrate that few commercial farms are seropositive for infection with influenza virus of the H7 subtype. The results from this study demonstrate that H7N9 virus was not detected at an expected prevalence of 30% for farms (with 20% expected prevalence within birds in the farm), 40% for wholesale vendors (30% within birds) and 50% for retail markets (30% within birds). Infection present below these levels may not have been detected. Finally, cloacal swabs were collected from poultry. It has been shown since this study was designed that influenza A(H7N9) virus was more likely to be detected in oropharyngeal swabs than cloacal [30] and this may have reduced the sensitivity of testing. Nevertheless environmental swabs were negative and these have been shown to be effective in detecting infected markets in other studies.
